# Supplementary material for: Artificial web of disclination lines in nematic liquid crystals
Source: Nat Commun. 2017 Aug 30;8:388. doi: 10.1038/s41467-017-00548-x (PMC5577038; doi:10.1038/s41467-017-00548-x)
Supplement: Supplementary file 1 — Supplementary Information [file 41467_2017_548_MOESM1_ESM.pdf]

Description of Supplementary Files

Title of file for HTML: Supplementary Information
Description: Supplementary Figures and Supplementary Notes

Title of file for HTML: Supplementary Movie 1
Description: Formation and stability of the web

Title of file for HTML: Supplementary Movie 2
Description: Transition between the square web and diagonal array of disclination lines

Title of file for HTML: Supplementary Movie 3
Description: Stability of the web of disclinations under variable angles

Title of file for HTML: Supplementary Movie 4
Description: 3D imaging of the web with fluorescent confocal microscopy

Title of file for HTML: Peer Review File
Description:

Supplementary Note 1: Structure and Interaction of Twist Disclination lines in Chiral Nematic Liquid Crystals

We derive the formulas that describe the director profile and the elastic interaction between twist disclination lines embedded in a cell of chiral nematic liquid crystals on the basis of the Frank theory of curvature elasticity[1]. We follow and extend the conformal mapping approach used by Geurst et al.[13] The case for achiral nematic liquid crystals can be treated as a special case by taking the limit of zero chirality. We only deal with the planar structure in which the director $\mathbf{n}$ is always constrained in the plane parallel to the flat cell surfaces:

$$\mathbf{n} = (n_x \quad n_y \quad n_z) = (0 \quad cos\varphi \quad sin\varphi)\,, \tag{1}$$

where the x-axis is taken normal to the surface and φ is the azimuthal angle of the director. The director lies in the $y - z$ plane. The cell has the thickness L and is infinitely extended in the $y - z$ plane.

The limit of this planar assumption has been numerically examined in Refs.[14] & [15], confirming the validity in the present case of nematics.

Governing Equations

For a given director field $\mathbf{n}(x, y, z)$, the Frank elastic free energy is given by

$$F = \int \tfrac{1}{2}K_{11}(\nabla\cdot\mathbf{n})^2 + \tfrac{1}{2}K_{22}(\mathbf{n}\cdot\nabla\times\mathbf{n} - q)^2 + \tfrac{1}{2}K_{33}(\mathbf{n}\times\nabla\times\mathbf{n})^2 + \tfrac{1}{2}K_{24}\nabla\cdot(\mathbf{n}\nabla\cdot\mathbf{n} + \mathbf{n}\times\nabla\times\mathbf{n})dv \tag{2}$$

where K_{11}, K_{22} and K_{33} are the elastic constants for splay, twist and bend deformations, respectively, K_{24} is the saddle-splay elastic constant, and q represents the natural twist that is nonzero only for chiral nematics. For a given boundary condition at the cell surface, the equilibrium director profile minimizes the total elastic free energy, and hence must satisfy the Euler-Lagrange equation.

For the planar structure, it is readily confirmed that the saddle-splay contribution identically vanishes. The Frank elastic free energy is reduced to:

$$F = \int \frac{1}{2}K_{22}\left(\frac{\partial\varphi}{\partial x} - q\right)^2 + \frac{1}{2}(K_{11}\sin^2\varphi + K_{33}\cos^2\varphi)\left(\frac{\partial\varphi}{\partial y}\right)^2 + \frac{1}{2}(K_{11}\cos^2\varphi + K_{33}\sin^2\varphi)\left(\frac{\partial\varphi}{\partial z}\right)^2 dv \tag{3}$$

or equivalently

$$F = \int \frac{1}{2}K_{22}\left(\frac{\partial\varphi}{\partial x} - q\right)^2 + \frac{1}{2}K\left[\left(\frac{\partial\varphi}{\partial y}\right)^2 + \left(\frac{\partial\varphi}{\partial z}\right)^2\right] - \frac{1}{2}\Delta K\cos2\varphi\left[\left(\frac{\partial\varphi}{\partial y}\right)^2 - \left(\frac{\partial\varphi}{\partial z}\right)^2\right] dv \tag{4}$$

where $K = (K_{11} + K_{33})/2$ and $\Delta K = (K_{11} - K_{33})/2$. We assume here that the splay and the bend elastic constants are equal, *i.e.* $K_{11} = K_{33} = K$ and $\Delta K = 0$, in order to make the equation analytically tractable. Then we obtain

$$F = \int_{-\infty}^{+\infty}\int_{-\infty}^{+\infty}\int_{-L/2}^{L/2} \frac{1}{2}K_{22}\left(\frac{\partial\varphi}{\partial x} - q\right)^2 + \frac{1}{2}K\left[\left(\frac{\partial\varphi}{\partial y}\right)^2 + \left(\frac{\partial\varphi}{\partial z}\right)^2\right] dxdydz \tag{5}$$

Introducing the scaled coordinates as

$$\tilde{x} = x, \qquad \tilde{y} = y\sqrt{\frac{K_{22}}{K}}, \qquad \tilde{z} = z\sqrt{\frac{K_{22}}{K}} \tag{6}$$

the Frank free energy can be rewritten as

$$F = \frac{1}{2}K\int_{-\infty}^{+\infty}\int_{-\infty}^{+\infty}\int_{-L/2}^{L/2}\left(\frac{\partial\varphi}{\partial\tilde{x}} - q\right)^2 + \left(\frac{\partial\varphi}{\partial\tilde{y}}\right)^2 + \left(\frac{\partial\varphi}{\partial\tilde{z}}\right)^2 d\tilde{x}d\tilde{y}d\tilde{z} \tag{7}$$

The equilibrium director profile is the one that minimizes F for a given boundary condition on the surface. The Euler-Lagrange equation now reads:

$$\frac{\partial^2\varphi}{\partial\tilde{x}^2} + \frac{\partial^2\varphi}{\partial\tilde{y}^2} + \frac{\partial^2\varphi}{\partial\tilde{z}^2} = 0 \tag{8}$$

which is the Laplace equation for φ in $(\tilde{x}, \tilde{y}, \tilde{z})$ space. The linearity of this equation allows superposition of solutions, which we will use extensively below. In particular, since a uniformly twisted state, represented in the form of $\varphi = q'\tilde{x} + \varphi_0$, satisfies the Laplace equation, it is allowed without loss of generality to set $q = 0$ and take account of the effect of natural twist separately.

Once the solution of the Laplace equation (satisfying the proper boundary condition) is known, the Free energy of the system can be evaluated as a surface integral over the boundary excluding the singularities, *i.e.* disclinations, as follows:

$$F = \frac{1}{2}K\int_V \left(\tilde{\nabla}\varphi\right)^2 d\tilde{v} = \frac{1}{2}K\int_V \tilde{\nabla}\cdot\left(\varphi\tilde{\nabla}\varphi\right) - \varphi\tilde{\Delta}\varphi \, d\tilde{v} = \frac{1}{2}K\int_{\partial V}\varphi\tilde{\nabla}\varphi\cdot d\tilde{\boldsymbol{S}} \tag{9}$$

where $\tilde{\nabla}$ and $\tilde{\Delta}$ stand for the gradient and the Laplace operators in $(\tilde{x}, \tilde{y}, \tilde{z})$ space, and use is made of Gauss' theorem to convert the volume to surface integral. If the solution is given as a sum of two independent solutions of the Laplace equation, $\varphi = \varphi_1 + \varphi_2$, the Frank free energy is given by

$$F = \frac{1}{2}K\int_{\partial V}\left(\varphi_1\tilde{\nabla}\varphi_1 + \varphi_2\tilde{\nabla}\varphi_2 + 2\varphi_1\tilde{\nabla}\varphi_2\right)\cdot d\tilde{\boldsymbol{S}} \tag{10}$$

where the first two terms are individual contributions from each solution and the last term gives the free energy of interaction.

Single Twist Disclination Line in $\pi/2$-twist Cell

We consider a straight twist disclination line running in parallel with the $\tilde{z}$-axis at $\tilde{x} = d$ and $\tilde{y} = 0$ as shown in Supplementary Figure 1. This disclination line is often referred to as a revers twist disclination, since it separates regions of inversely twisted states. The solution of the Laplace equation for this *two dimensional* geometry can be obtained by using a type of conformal mapping called the Schwarz-Christoffel transformation:

$$\boldsymbol{Z}_2 = \sin\pi\frac{\boldsymbol{Z}_1}{L} \tag{11}$$

which transforms the infinite half strip ($\boldsymbol{Z}_1 = \tilde{x} + i\tilde{y}, -\frac{L}{2} \leq \tilde{x} \leq \frac{L}{2}, 0 \leq \tilde{y}(0 \geq \tilde{y})$) on the complex $\boldsymbol{Z}_1$ plane to the entire upper(lower) half plane of the complex $\boldsymbol{Z}_2$ plane. By applying this transformation to a trivial harmonic function, satisfying the required boundary conditions,

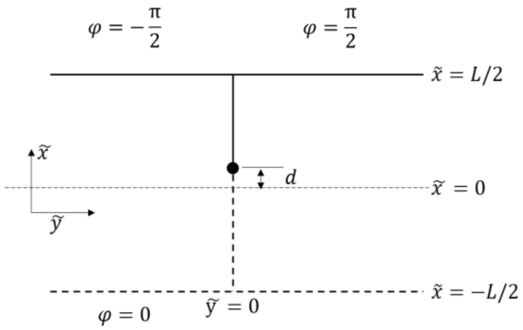

Supplementary Figure 1: Geometry of single twist disclination line and the boundary conditions. The disclination line located at $\tilde{x} = d$, running along the z-axis separates the regions of oppositely twisted states.

$$\varphi = -\frac{1}{2}\mathrm{Im}\left\{\ln\left(\boldsymbol{Z}_2 - \sin\frac{\pi d}{L}\right)\right\} + \begin{cases} +\frac{\pi}{2} & \mathrm{Re}(\boldsymbol{Z}_2) > 0 \\ -\frac{\pi}{2} & \mathrm{Re}(\boldsymbol{Z}_2) < 0 \end{cases} \tag{12}$$

we immediately obtain the solution:

$$\varphi(\tilde{x}, \tilde{y}, \tilde{z}: d) = \frac{1}{2}\left[\tan^{-1}\left(\frac{\sin\frac{\pi\tilde{x}}{L}\cosh\frac{\pi\tilde{y}}{L} - \sin\frac{\pi d}{L}}{\cos\frac{\pi\tilde{x}}{L}\sinh\frac{\pi\tilde{y}}{L}}\right) + \frac{\pi}{2}\mathrm{sgn}(\tilde{y})\right] \tag{13}$$

On using the scaling Supplementary Equation 6 back in this equation, we obtain Eq.(1). The minus sign in Eq.(1) corresponds to the reversal of the sense of twist, thereby, reversing the handedness of the twist disclination line, which is equivalent to $\varphi(\tilde{x}, -\tilde{y}, \tilde{z}: d) = -\varphi(\tilde{x}, \tilde{y}, \tilde{z}: d)$.

Free Energy of Single Twist Disclination Line in $\pi/2$-twist Cell

Substituting Supplementary Equation 13 into Supplementary Equation 9, we obtain the surface integral representation of the free energy. Since $\varphi = 0$ over the lower surface, we only need to calculate the contributions from the upper surfaces (S_1, S_5), the two vertical plane surfaces (S_2, S_4) and the cylindrical surface (S_3) encircling the disclination line as depicted in Supplementary Figure 2 by solid blue lines.

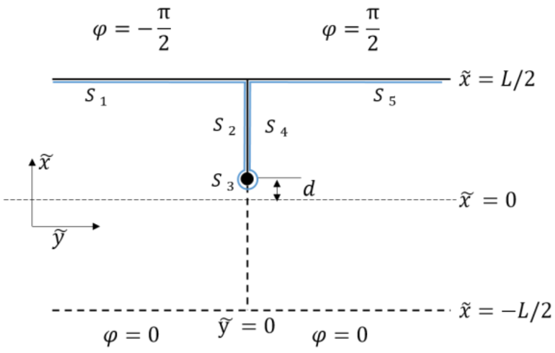

Supplementary Figure 2: Surface integration for free energy calculation. The blue line indicates the surface over which the integration should be performed.

Using

$$\left(\frac{\partial\varphi}{\partial\tilde{x}}\right)_{\tilde{x}=L/2} = \frac{\pi}{2L}\frac{\sinh\frac{\pi\tilde{y}}{L}}{\cosh\frac{\pi\tilde{y}}{L} - \sin\frac{\pi d}{L}} \tag{14}$$

into Supplementary Equation 9, we obtain as the contribution from (S_1, S_5) surfaces as

$$\begin{aligned} F_{1,5} &= \frac{1}{2}K\int_{S_1,S_5}\varphi\tilde{\nabla}\varphi\cdot d\tilde{\boldsymbol{S}} = \frac{1}{2}K\int\left\{\int_0^\infty \frac{\pi^2}{2L}\frac{\sinh\frac{\pi\tilde{y}}{L}}{\cosh\frac{\pi\tilde{y}}{L} - \sin\frac{\pi d}{L}}d\tilde{y}\right\}d\tilde{z} \\ &= \int\left\{\frac{\pi}{4}K\lim_{\tilde{y}\to\infty}\ln\left(\cosh\frac{\pi\tilde{y}}{L} - \sin\frac{\pi d}{L}\right) - \frac{\pi}{4}K\ln\left(1 - \sin\frac{\pi d}{L}\right)\right\}d\tilde{z} \end{aligned} \tag{15}$$

The contributions from (S_2, S_4) surfaces are calculated by using

$$\frac{\partial\varphi}{\partial\tilde{y}} = -\frac{\pi}{2L}\frac{\sin\frac{\pi\tilde{x}}{L} - \sin\frac{\pi d}{L}\cosh\frac{\pi\tilde{y}}{L}}{\left(\sin\frac{\pi\tilde{x}}{L} - \sin\frac{\pi d}{L}\cosh\frac{\pi\tilde{y}}{L}\right)^2 + \cos^2\frac{\pi d}{L}\sinh^2\frac{\pi\tilde{y}}{L}}\cos\frac{\pi\tilde{x}}{L} \tag{16}$$

$$\left(\frac{\partial\varphi}{\partial\tilde{y}}\right)_{\tilde{y}=0} = -\frac{\pi}{2L}\frac{\cos\frac{\pi\tilde{x}}{L}}{\sin\frac{\pi\tilde{x}}{L} - \sin\frac{\pi d}{L}} \tag{17}$$

to be

$$F_{2,4} = \frac{1}{2}K\int_{S_2,S_4}\varphi\tilde{\nabla}\varphi\cdot d\tilde{\mathbf{S}} = \frac{1}{2}K\int\left\{\int_{d+\delta}^{L/2}\frac{\pi^2}{2L}\frac{\cos\frac{\pi\tilde{x}}{L}}{\sin\frac{\pi\tilde{x}}{L}-\sin\frac{\pi d}{L}}d\tilde{x}\right\}d\tilde{z} = \int\left\{\frac{\pi}{4}K\ln\frac{1-\sin\frac{\pi d}{L}}{\sin\frac{\pi(d+\delta)}{L}-\sin\frac{\pi d}{L}}\right\}d\tilde{z}$$
$$\approx \int\left\{\frac{\pi}{4}K\ln\frac{1-\sin\frac{\pi d}{L}}{\frac{\pi\delta}{L}\cos\frac{\pi d}{L}}\right\}d\tilde{z} \tag{18}$$

where δ is the cutoff length due to the finite core size on the order of 10nm. Finally, the contribution from the surface (S_3) vanishes since $\tilde{\nabla}\varphi$ is tangential to the surface at sufficiently close to the core. Summing all these contributions and applying the scaling Supplementary Equation 6, we obtain the free energy per unit depth along the z-axis

$$F = F_{1,5} + F_{2,4} = \frac{\pi}{4}\sqrt{KK_{22}}\lim_{\tilde{y}\to\infty}\ln\left(\cosh\frac{\pi\tilde{y}}{L}-\sin\frac{\pi d}{L}\right)+\frac{\pi}{4}\sqrt{KK_{22}}\ln\frac{L}{\pi\delta\cos\frac{\pi d}{L}}$$
$$\to \lim_{y\to\infty}\frac{\pi^2}{4L}K_{22}y+\frac{\pi}{4}\sqrt{KK_{22}}\ln\frac{L}{\pi\delta\cos\frac{\pi d}{L}} \tag{19}$$

where the first term is the deformation energy of a uniform π/2-twisted layer of width $2y$. Subtracting this bulk twist contribution from the total free energy, we are left with the second term that gives the excess free energy or the line tension associated with the twist disclination line Eq.(2).

Interaction Force between Two Parallel Twist Disclination Lines

A. Vertical Arrangement

The director profile for the two parallel disclination line geometry as depicted in Supplementary Figure 3 is given by the superposition of the solutions for individual lines given by Supplementary Equation 13:

$$\varphi(\tilde{x},\tilde{y},\tilde{z}:d_1,d_2) = \varphi(\tilde{x},\tilde{y},\tilde{z}:d_1)\pm\varphi(\tilde{x},\tilde{y},\tilde{z}:d_2) \tag{20}$$

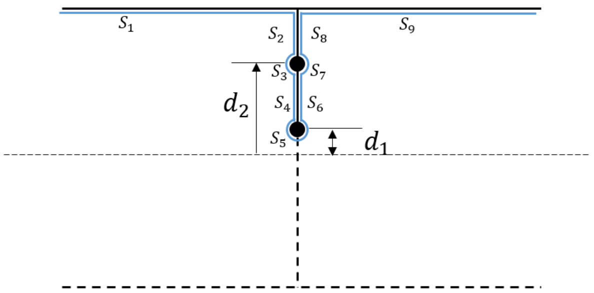

Supplementary Figure 3: Two vertically separated parallel twist disclination lines. The blue line indicates the surface over which the integration should be performed.

where the plus and minus signs refer to the same and the opposite handedness of the disclination lines.
The interaction free energy between two parallel disclination lines can be calculated by integrating the coupling term in Supplementary Equation 10 over the surfaces S_1 through S_9:

$$\Delta F = \pm\frac{1}{2}K\int_{\partial V}2\varphi(d_1)\tilde{\nabla}\varphi(d_2)\cdot d\tilde{\mathbf{S}} \tag{21}$$

Integration over S_1, S_2, S_8 and S_9 is essentially identical to Supplementary Equation 15 and Supplementary Equation 19 except for the plus and minus signs. Integration over S_3, S_5 and S_7 vanishes. Per unit depth, the interaction free energy for $d_2 > d_1$ is written as

$$\Delta F_{4,6} = \pm\frac{1}{2}\sqrt{KK_{22}}\int_{d_1+\delta}^{d_2-\delta}\frac{\pi^2}{L}\frac{\cos\frac{\pi\tilde{x}}{L}}{\sin\frac{\pi\tilde{x}}{L}-\sin\frac{\pi d_2}{L}}d\tilde{x} = \pm\frac{\pi}{2}\sqrt{KK_{22}}\ln\frac{\frac{\pi\delta}{L}\cos\frac{\pi d_2}{L}}{\sin\frac{\pi d_2}{L}-\sin\frac{\pi d_1}{L}} \tag{22}$$

Adding up all the contributions, we obtain the free energy per unit depth associated with the vertically separated disclination line pair as

$$F = F_{bulk}^{\pm} + \frac{\pi}{4}\sqrt{KK_{22}}\ln\frac{L}{\pi\delta\cos\frac{\pi d_1}{L}} + \frac{\pi}{4}\sqrt{KK_{22}}\ln\frac{L}{\pi\delta\cos\frac{\pi d_2}{L}} \pm \frac{\pi}{4}\sqrt{KK_{22}}\ln\left(\sin\frac{\pi d_2}{L} - \sin\frac{\pi d_1}{L}\right)^{-2} \tag{23}$$

where the plus and minus signs refer to the same and the opposite handedness of the disclination lines. $F_{bulk}^{\pm}$ is the bulk twist deformation energy, which is specifically $F_{bulk}^{-} = 0$ and $F_{bulk}^{+} = \lim_{y\to\infty}\frac{\pi^2}{L}K_{22}y$. The last term of Supplementary Equation 23 is the interaction energy between twist disclination lines given in Eq.(3).

Supplementary Figure 4 shows the free energy landscape over the (d_1, d_2) plane for the cases of the same and the opposite handedness of disclinations. When the disclinations are of the same handedness, the minimum occurs at

$$d_1 = -\frac{L}{4} \qquad d_2 = \frac{L}{4} \tag{24}$$

as a result of force balance between the disclination-disclination repulsion and the surface-disclination repulsion. When the disclination lines have opposite handedness, on the other hand, the disclination-disclination interaction is strongly attractive toward $d_1 = d_2$, and there appears a deep minimum at the middle of the cell.

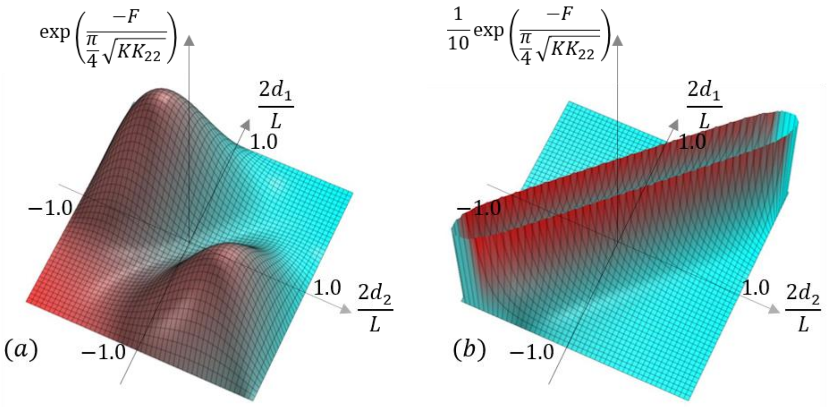

Supplementary Figure 4: Free energy landscape as in terms of the probability density. (a) Disclinations of the same handedness, (b) Disclinations of the opposite handedness.

B. General Arrangement

We now consider a general case of two parallel disclination lines that are laterally as well as vertically separated as shown in Supplementary Figure 5. The solution is again given by the superposition of the contributions from each individual disclination:

$$\varphi(\tilde{x}, \tilde{y}, \tilde{z}: d_1, d_2) = \varphi(\tilde{x}, \tilde{y}, \tilde{z}: d_1) \pm \varphi(\tilde{x}, \tilde{y} - h, \tilde{z}: d_2) \tag{25}$$

where the plus and minus signs refer to the same and the opposite handedness of the disclination lines. Taking the same procedure as above and using Supplementary Equation 16, the surface integrals

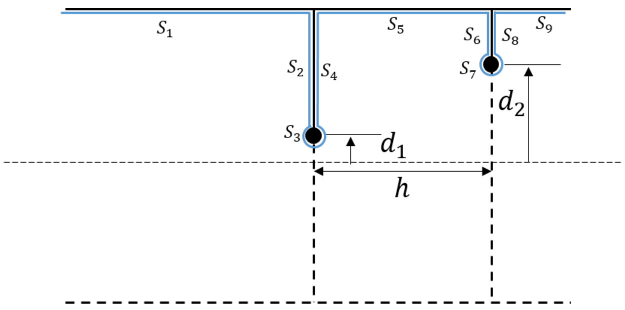

Supplementary Figure 5: The case of two parallel twist disclination lines at arbitrary positions. The blue line indicates the surface over which the integration should be performed.

can be made in a closed form to replace Supplementary Equation 23 with the following equation:

$$F = F_{bulk}^{\pm} + \frac{\pi}{4}\sqrt{KK_{22}}\ln\frac{L}{\pi\delta\cos\frac{\pi d_1}{L}} + \frac{\pi}{4}\sqrt{KK_{22}}\ln\frac{L}{\pi\delta\cos\frac{\pi d_2}{L}}$$
$$\pm\frac{\pi}{4}\sqrt{KK_{22}}\ln\left\{\sinh^2\sqrt{\frac{K_{22}}{K}}\frac{\pi}{L}h + 2\left(1-\cosh\sqrt{\frac{K_{22}}{K}}\frac{\pi}{L}h\right)\sin\frac{\pi d_2}{L}\sin\frac{\pi d_1}{L} + \left(\sin\frac{\pi d_2}{L} - \sin\frac{\pi d_1}{L}\right)^2\right\}^{-1}$$

(26)

It follows from this expression of the free energy per unit length of disclinations that the disclinations of the same (opposite) handedness repel (attract) each other irrespective of their relative arrangement inside the uniform twist cell. A notable difference from the vertical arrangement is the presence of a long range (infinite range, in fact) interaction associated with the twist deformations in the h–wide region between the disclination lines. In the limit of large h, the last term of Supplementary Equation 26 is dominated by $\sinh^2\sqrt{\frac{K_{22}}{K}}\frac{\pi}{L}h$ contribution, and it approaches

$$\pm\frac{\pi}{4}\sqrt{KK_{22}}\ln\left\{\sinh^2\sqrt{\frac{K_{22}}{K}}\frac{\pi}{L}h\right\}^{-1} \rightarrow \mp\frac{\pi^2}{2L}K_{22}h$$

(27)

indicating that there is a gain (loss) of deformation energy of π-twisted state as h is increased, when the disclinations have the same (opposite) handedness. As a result, the region between the disclination lines become untwisted in the case of same handedness, whereas in the opposite handedness case, only this area becomes π-twisted inside the untwisted background. Twist disclination lines of the same handedness in a uniform twist cell, therefore, keep separating until the boundary prevents their further movement or they meet disclination lines of the opposite handedness and disappear. This, in fact, is one of the reasons why it is practically difficult to have stable network of disclination lines.

Interaction Force between Obliquely Oriented Twist Disclination Lines

Of particular interest is the interaction forces between disclination lines when they are intersecting at an oblique angle Φ as illustrated in Supplementary Figure 6. In this case, interaction forces act only in the vicinity of the intersection within the range of extra elastic distortions around the disclination, roughly measured by $L\sqrt{K/K_{22}}$. Unlike the case of parallel lines, the force is localized, giving rise to a deformation of the disclination line from the straight shape. Here, we neglect this line deformation based on an assumption that the line tension is sufficiently high to keep the straight linear shape.

Since the Frank elastic energy is isotropic in the $y-z$ plane, *i.e.* an arbitrary constant can be added to φ without influencing the free energy, we can always choose one disclination line passing $\tilde{x} = d_1, \tilde{y} = 0$ to be parallel to the z axis and is described by $\varphi_1 = \varphi(\tilde{x}, \tilde{y}: d_1)$, which is independent of z. The second disclination line passes $\tilde{x} = d_2, \tilde{y} = 0$, and is rotated by the angle Φ. The director profile for the rotated disclination line is written as $\varphi_2 =$

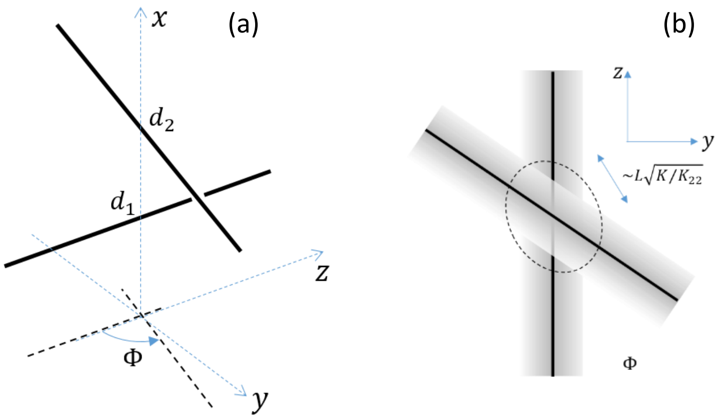

Supplementary Figure 6: Two disclination lines intersecting at an oblique angle. (**a**) Perspective view of the intersecting lines. (**b**) Top view of the intersection showing the locally extended areas of interaction.

$\varphi(\tilde{x}, \tilde{y}\cos\Phi + \tilde{z}\sin\Phi: d_2)$. Note that when $\Phi = \pi$, the handedness of the disclination line is reversed. Superposing φ_1 and φ_2, we obtain the solution for the oblique case as

$$\varphi(\tilde{x}, \tilde{y}, \tilde{z}: d_1, d_2, \Phi) = \varphi(\tilde{x}, \tilde{y}, \tilde{z}: d_1) + \varphi(\tilde{x}, \tilde{y}\cos\Phi + \tilde{z}\sin\Phi: d_2) \tag{28}$$

The interaction energy is then given by

$$\Delta F = \frac{1}{2}K \int_{\partial V} 2\varphi(\tilde{x}, \tilde{y}: d_1)\tilde{\nabla}\varphi(\tilde{x}, \tilde{y}\cos\Phi + \tilde{z}\sin\Phi: d_2) \cdot d\tilde{\boldsymbol{S}} \tag{29}$$

The surface of integration is the one encircling the first disclination as shown in Supplementary Figure 2. On the surfaces (S_2, S_4) (see Supplementary Figure 2), the surface normal has only the $\tilde{y}$-component, so the above equation can be reduced to

$$\Delta F = \pi K\cos\Phi \int \left\{ \int_{d_1+\delta}^{L/2} \left(\frac{\partial\varphi(\tilde{x}, \tilde{y}: d_2)}{\partial\tilde{y}} \right)_{\tilde{y}=\tilde{z}\sin\Phi} d\tilde{x} \right\} d\tilde{z} \tag{30}$$

Referring to the explicit formula for the $\tilde{y}$ derivative of φ, Supplementary Equation 16, it is readily understood that it is peaked at $\tilde{y} = 0$ and decays exponentially with the characteristic length L ($L\sqrt{K/K_{22}}$ for y). Therefore, the integrand can make an appreciable contribution only within a narrow region of the size of $L/\sin\Phi$ about the intersection. This equation indicates that, as the disclination line pair with the same handedness is gradually squeezed from parallel (vertical) configuration, there is a continuous crossover from repulsive to attractive regime according to $\cos\Phi$. At the orthogonal arrangement, the direct interaction force disappears. Unless the angle of intersection is very close to 0 or π, the interaction is practically localized in the vicinity of the intersection.

Interaction of Twist Disclination line with Surface Pattern

We confine our attention to the case in which a non-singular solution $\varphi_i(\tilde{x}, \tilde{y}, \tilde{z})$ exists, satisfying the given boundary condition on the surface. The periodic orientational pattern shown in Supplementary Figure 7, which is the constituent of the squared pattern used in the present experiments, allows a non-singular solution of the Laplace equation as

$$\varphi_i(\tilde{x}, \tilde{y}, \tilde{z}) = \frac{\pi}{p}\tilde{y}\sqrt{\frac{K}{K_{22}}}\left(\frac{1}{2} - \frac{\tilde{x}}{L}\right) \tag{31}$$

where p stands for the pitch. This solution corresponds to the cell structure in which the orientation at the top surface at $\tilde{x} = L/2$ is $\varphi = 0$, and the periodic pattern with increasing azimuthal angle is imposed on the bottom surface. Being linear in both $\tilde{x}$ and $\tilde{y}$, it trivially satisfies the Laplace equation. Superposing the singular solution, we obtain

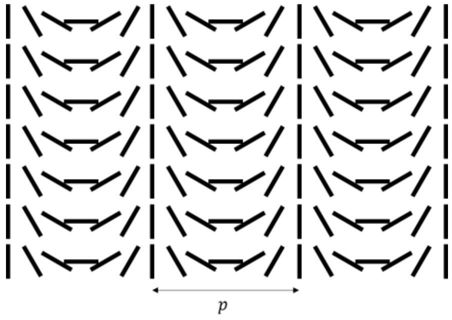

Supplementary Figure 7: Top view of the periodic orientational pattern on the bottom surface with a linearly increasing azimuhtal angle. For each pitch, the director makes a 180 degrees rotation.

$$\varphi_T(\tilde{x}, \tilde{y}: d) = \varphi(\tilde{x}, \tilde{y}: d) + \varphi_i(\tilde{x}, \tilde{y}, \tilde{z}) \tag{32}$$

that describes a single twist disclination line confined in a cell with the periodic surface pattern. The coupling free energy between the disclination and the surface pattern is given by

$$\Delta F = \frac{1}{2}K \int_{\partial V} 2\varphi(\tilde{x}, \tilde{y}: d)\tilde{\nabla}\varphi_i(\tilde{x}, \tilde{y}, \tilde{z}) \cdot d\tilde{\boldsymbol{S}} \tag{33}$$

Extracting only the part that depends on the position of the disclination, we obtain

$$\Delta F = \frac{1}{2} K \int \left\{ \int_d^{L/2} -2\pi \frac{\pi}{p} \sqrt{\frac{K}{K_{22}}} \left(\frac{1}{2} - \frac{\tilde{x}}{L} \right) d\tilde{x} \right\} d\tilde{z} = \int \left\{ \int_d^{L/2} -\frac{\pi^2}{2p} K \left(1 - 2\frac{\tilde{x}}{L} \right) d\tilde{x} \right\} dz \tag{34}$$

The free energy per unit z depth (or unit length of disclination line) is given by

$$\Delta F_u = -\frac{\pi^2}{2p} K \left(\frac{L}{4} - d + \frac{d^2}{L} \right) \tag{35}$$

From this result follows the force that the surface pattern at the bottom surface exerts on the disclination line as

$$f_p = -\frac{\partial \Delta F_u}{\partial d} = -\frac{\pi^2}{2p} K \left(1 - 2\frac{d}{L} \right) \tag{36}$$

This indicates that the periodic pattern with an increasing azimuth in the direction consistent with the handedness of the disclination line pulls the disclination line toward the pattern. When the disclination has the opposite handedness, the force acts in the opposite direction with the same magnitude. As a result, when both bottom and top surfaces have the periodic pattern in the same sense as illustrated in Supplementary Figure 8, the total force acting on the disclination line from both surfaces becomes position independent:

$$f_p = -\frac{\pi^2}{p} K \tag{37}$$

The direction of the force is opposite for a disclination of the opposite handedness.

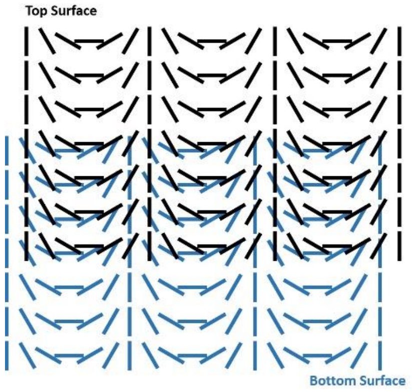

Supplementary Figure 8: Identical surface orientational patterns overlapped. This configuration creates a position independent force on twist disclination lines.

It is of interest to consider the balance of force in the case of parallel disclination lines of opposite handedness in the presence of the surface patterns. It has been shown that the disclination lines attract each other, and are expected to eventually annihilate themselves in a uniform cell. To see how the pattern generated force could counter this trend, we set $-d_1 = d_2 \equiv d$ in Supplementary Equation 23 and calculate the attractive force on each line:

$$f_a = -\frac{1}{2} \frac{\partial}{\partial d} \left\{ \frac{\pi}{2} \sqrt{K K_{22}} \ln \left(\tan \frac{\pi d}{L} \right) \right\} = -\frac{\pi^2}{2L} \sqrt{K K_{22}} \frac{1}{\sin 2 \frac{\pi d}{L}} \tag{38}$$

The smallest attractive force $\frac{\pi^2}{2L}\sqrt{KK_{22}}$ occurs at $d = L/4$; if the *pulling* force due to surface pattern is larger than the smallest attractive force, there appears a metastable position for the disclination line. Since the attractive force is inversely proportional to the cell thickness, while the force due to surface pattern is independent of the thickness, there is a threshold thickness of the cell L_{th} below which the disclination line pair of opposite handedness is always unstable for ultimate annihilation:

$$L_{th} = \tfrac{1}{2} p \sqrt{K_{22}/K} \tag{39}$$

In case the surface patterns have the increasing azimuth in the opposite directions as shown in Supplementary Figure 9, the force from the top surface is written as

$$f_p = \frac{\pi^2}{2p} K \left(1 + 2\frac{d}{L}\right) \tag{40}$$

Combining the contributions from both surface, we now obtain

$$f_p = 2\frac{\pi^2 d}{pL} K \tag{41}$$

indicating that the force changes sign, passed the mid plane of the cell, and hence is always toward the closer surface. If the handedness of the disclination line is reversed (although this arrangement seems unlikely in practice), it becomes incompatible with both surfaces and is subjected to the opposite force:

$$f_p = -2\frac{\pi^2 d}{pL} K \tag{42}$$

which is always toward the mid plane of the cell.

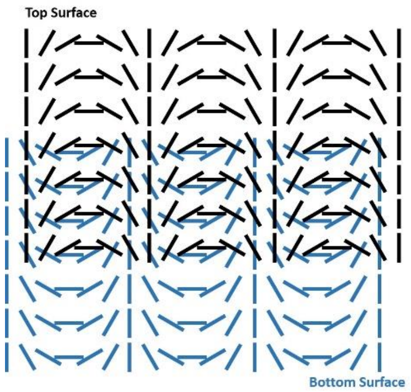

Supplementary Figure 9: Opposite surface orientational patterns overlapped. This configuraton creates a bidirectional force attracting the disclination lines toward the closer surface.

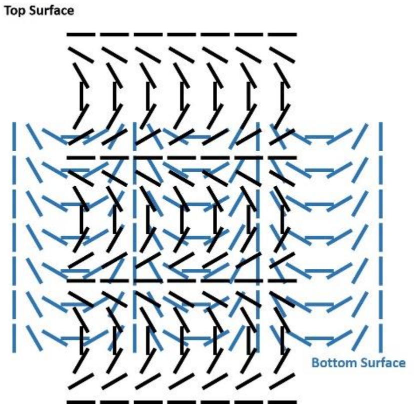

Supplementary Figure 10: Orthogonally oriented surface patterns. Orientations on the top substrate are given in black and those on the bottom substrate are in blue.

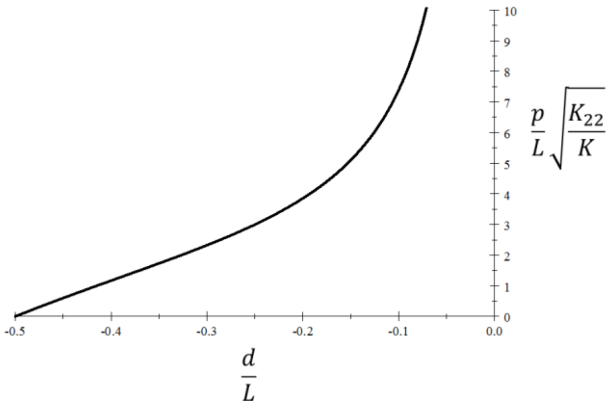

Supplementary Figure 11: Stable separation of the straight disclination line from the mid plane of the cell for the orthogonal surface patterns. The stable separation d is given as a function of the pitch of the surface pattern p.

Finally, let us consider the case of orthogonal surface patterns as shown in Supplementary Figure 10, since this is especially relevant for the web of disclination lines reported here. The azimuthal angle at the top surface increases in z direction as $\pi z/p$. Referring to Supplementary Equation 31, we see that the force between the disclination line running in the y direction and the top surface vanishes. Since the interaction between orthogonally crossing disclination lines is also vanishing, we only need to consider the balance of force on the disclination line along the y-axis, Supplementary Equation 34, and the force due to line tension, Supplementary Equation 19. The stable z position of the disclination line is then given by

$$\frac{p}{L}\sqrt{\frac{K_{22}}{K}} = 2\frac{1 - \frac{d}{L}}{\tan\frac{\pi d}{L}} \tag{43}$$

which is graphically represented in Supplementary Figure 11. Even for a fairly large pitch of the pattern p relative to the cell thickness, there remains an appreciate distance from the middle of the cell. This is consistent with the experimental observations.

Effect of Natural Twist

As long as the planar structure is maintained, the contribution of the natural twist ($q \neq 0$) is to add a null Lagrangian. Getting back to the starting equation of free energy, Supplementary Equation 7, we find

$$F = \frac{1}{2}K\int_{-\infty}^{+\infty}\int_{-\infty}^{+\infty}\int_{-\frac{L}{2}}^{\frac{L}{2}}\left(\frac{\partial\varphi}{\partial\tilde{x}} - q\right)^2 + \left(\frac{\partial\varphi}{\partial\tilde{y}}\right)^2 + \left(\frac{\partial\varphi}{\partial\tilde{z}}\right)^2 d\tilde{x}d\tilde{y}d\tilde{z}$$
$$= \frac{1}{2}K\int_{-\infty}^{+\infty}\int_{-\infty}^{+\infty}\int_{-\frac{L}{2}}^{\frac{L}{2}}\left(\frac{\partial\varphi}{\partial\tilde{x}}\right)^2 + \left(\frac{\partial\varphi}{\partial\tilde{y}}\right)^2 + \left(\frac{\partial\varphi}{\partial\tilde{z}}\right)^2 d\tilde{x}d\tilde{y}d\tilde{z}$$
$$+ \frac{1}{2}K\int_{-\infty}^{+\infty}\int_{-\infty}^{+\infty}\left\{\frac{1}{2}Kq^2L - 2q(\varphi(L/2) - \varphi(-L/2))\right\}d\tilde{y}d\tilde{z}$$
(44)

Hence, as long as the boundary conditions at the surfaces, $\varphi(L/2), \varphi(-L/2)$, are fixed, the natural twist locally provides a constant bias of free energy, so that the analytical treatments and results so far obtained remain formally valid. However, the stability of a particular configuration of the director is subjected to change, because the natural twist breaks the energetic symmetry of oppositely twisted states.

With a finite natural twist, an energetically stable twist disclination line can occur between the regions twisted by $\pi/2 + qL$ and $-\pi/2 + qL$ as illustrated in Supplementary Figure 10. So, the director orientations at the surfaces are not necessarily orthogonal at the point of disclination line. The explicit solution for chiral cases can be readily obtained by superposition of natural twist on top of the achiral solution we have already obtained. Let $\varphi_a(x, y, z)$ be the solution for achiral system, then the solution for the chiral system is written as

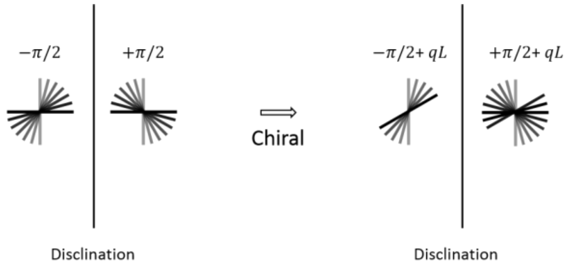

Supplementary Figure 12: Chirality effect in the stability of twist disclination line. In the achiral system (left), the twist is opposite yet is of the same magnitude across the disclination line. In chiral systems (right), the twist angle is no longer the same, but suffers a constant bias due to the presence of natural twist.

$$\varphi = \varphi_a(x, y, z) + qx + \varphi_0$$
(45)

where φ_0 is an arbitrary constant. Since $qx + \varphi_0$ satisfies the Laplace equation, the above equation is also the solution of the Laplace equation. Unlike the achiral nematic, the director profile right above and below the disclination line is not uniform, but is a twisted state as required by the natural twisting power. For these reasons, the interpretation of polarizing microscope observation cannot be as straightforward as in the case of achiral systems.

We can generalize the above argument further to include arbitrary, yet uniform boundary conditions. To satisfy the boundary condition, we can add more twist in such a way that the boundary conditions are met:

$$\varphi = \varphi_a(x, y, z) + qx + \alpha x + \varphi_0 \quad (46)$$

The constants α and φ_0 must be chosen to satisfy the given boundary conditions. Due to the equivalence of states by an integral multiple of π, the choice of these constants is not unique.

When $\alpha \neq 0, n\pi$, a straight twist disclination line is not in mechanical equilibrium with the surrounding. The imbalance of elastic free energy on both sides of the disclination line induces a bending of the line. Due to the

isotropy on the plane, the curvature of bending of the disclination line can be written in the form of Young-Laplace equation:

$$\frac{\gamma}{R} = \Delta f \tag{47}$$

where γ is the line tension of disclination Eq.(2) and Supplementary Equation 19, R is the radius of curvature, and Δf is the difference of elastic energy density across the disclination line. The center of curvature must be taken in the side with lower elastic energy, noting that the region of lower elastic energy tends to expand. Assuming $\gamma \sim 10K$ and $\Delta f \sim K/L$, the radius of curvature is roughly estimated to be $R \sim 10L$, which appears to justify the assumption of straight disclination lines in calculating the interaction forces at the intersection.

Consideration on Anisotropic Elastic Constants $K_{11} \neq K_{33}$

The validity of the superposition approach we have extensively used rests on the in-plane isotropy of the system resulting from $K_{11} = K_{33}$. In case $\Delta K \neq 0$ in Supplementary Equation 4, the $\cos 2\varphi$ term makes the Euler-Lagrange equation nonlinear, and the superposition is no longer valid. According to existing data of the elastic constants of calamitic liquid crystals, $1 < K_{33}/K_{11} < 2$ and in the majority of cases, $1 < K_{33}/K_{11} < 1.5$ is satisfied. Even if we assume $K_{33}/K_{11} = 1.5$, we find $\Delta K/K = 0.2$, and it may be permitted to treat ΔK term as a small correction in most practical cases.

Deviation from the in-plane isotropy due to $\Delta K \neq 0$ should manifest in the presence of preferred orientation of disclination lines relative to the base surface alignment, or in terms of the orientation dependence of radius of curvature of a disclination line, Supplementary Equation 47, when subjected to unbalanced twist deformations.

Supplementary Note 2: Distribution of Twist Deformation Energy on the Web of Disclination Lines

The 3D structure of director distributions in the surface patterned cell is not straightforward. As an aid for better appreciation of the director profile and the twist energy distribution, graphical representation of the energy distribution is presented below in relation to the stability of the square web of disclination lines and the competing array of diagonal disclination lines. The diagonal lines are energetically more stable than the square web; however, the energy and topological barrier for the transformation of the square web to the diagonal array is sufficiently high, so that the "metastable" square web can be easily formed and maintained indefinitely without noticeable relaxation to the diagonal form.

In Supplementary Figure 13 shown is the projection of the director orientation imposed on the twist energy density map. The distribution of twist angles in a unit cell is written as

$$\varphi_t = \frac{\pi}{p}(z - y) \tag{48}$$

As shown in Supplementary Figure 14, along the diagonal lines from bottom left to top right, the twist angle is constant. In the square web, the disclination lines are so formed that the intersections of the disclination lines (lattice points) are anchored on the zero twist diagonal. Then, the quadrants of the intersection have either 0 or $\pm\pi$ twist. Since the twist energy makes a jump across the disclination line except at the mid points of the lattice where the twist angle is $\pi/2$ on

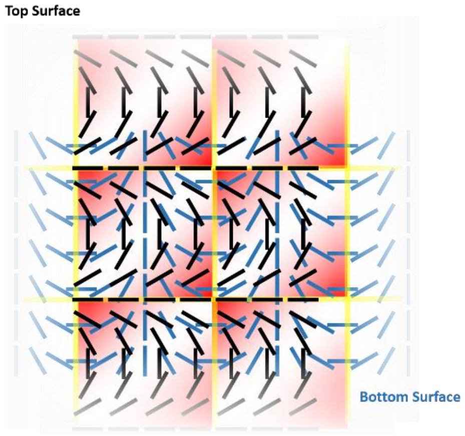

Supplementary Figure 13: Orthogonal orientational surface patterns superimposed on the twist energy density map. The gradation in red indicates the magnitude of twist energy density, and the yellow lines are twist disclination lines forming the web.

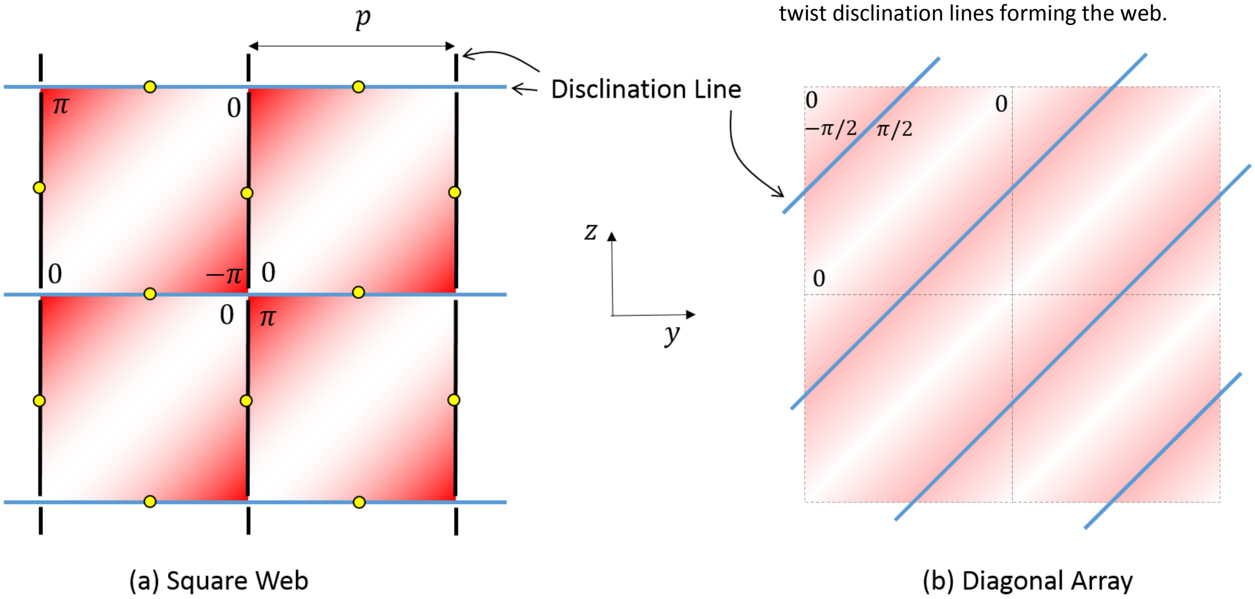

Supplementary Figure 14: Twist energy density map on the square web and the diagonal array of disclination lines. (a) Square web of disclination lines consisting of the upper (blue) and the lower (black) disclination lines. The yellow dots at the center of the edge indicate the position where the twist angles are $\pm\pi/2$. (b) Distribution of twist angles in the case of diagonal array of disclination lines (blue). Note that the regions of twist angle larger than $\pi/2$ are converted to lower twist states.

both sides of the line, the straight disclination lines cannot be in mechanical equilibrium, but must be curved. As we have already discussed, this effect can be practically negligible unless the pitch p is too large compared to the cell thickness. It is therefore seen that the disclination lines are anchored at the corner and the middle point of the square lattice. Although metastable, the intersecting disclination lines are separated in the depth direction by the forces from the surface and the pattern.

A more energetically stable state of disclination lines is the diagonal array shown in Supplementary Figure 14b. The disclination lines are running diagonally connecting the middle points of the lattice (there is no longer a lattice, though) where the twist angle is $\pm\pi/2$. Across the disclination line, the twist angle jumps from $+\pi/2$ to $-\pi/2$, yet the elastic energy are the same on both sides. Therefore, the straight diagonal lines are in mechanical equilibrium. Furthermore, the higher twist energy areas bearing twist angles between $\pi/2$ and π are converted into lower twisted state not exceeding $\pi/2$. In addition, the total length of the diagonal lines to span the entire space of pattern is shorter by a factor of $1/\sqrt{2}$ than the total length of lines in the square web. For these reasons, the diagonal array is energetically more stable. Nevertheless, the kinetic process we employed to fabricate the square web as well as the energy and topological barrier separating these states is effective enough to keep the metastable square web of disclinations intact for long period of time.

Shown in Supplementary Figure 15 is the coexistence of square web and diagonal disclination lines. This is a magnified view of the left bottom part of the overlapped region. The twist angle distribution is given by

$$\varphi_t = -\frac{\pi}{p}(z + y) \tag{49}$$

Hence the lines of constant twist angle are diagonal from the right bottom to the left top; the mirror image of that in Supplementary Figure 14. In the middle of the picture, a two lattice wide row of square web has been converted to an array of diagonal lines. The diagonal lines did not invade the square web any further in the time frame of experiment.

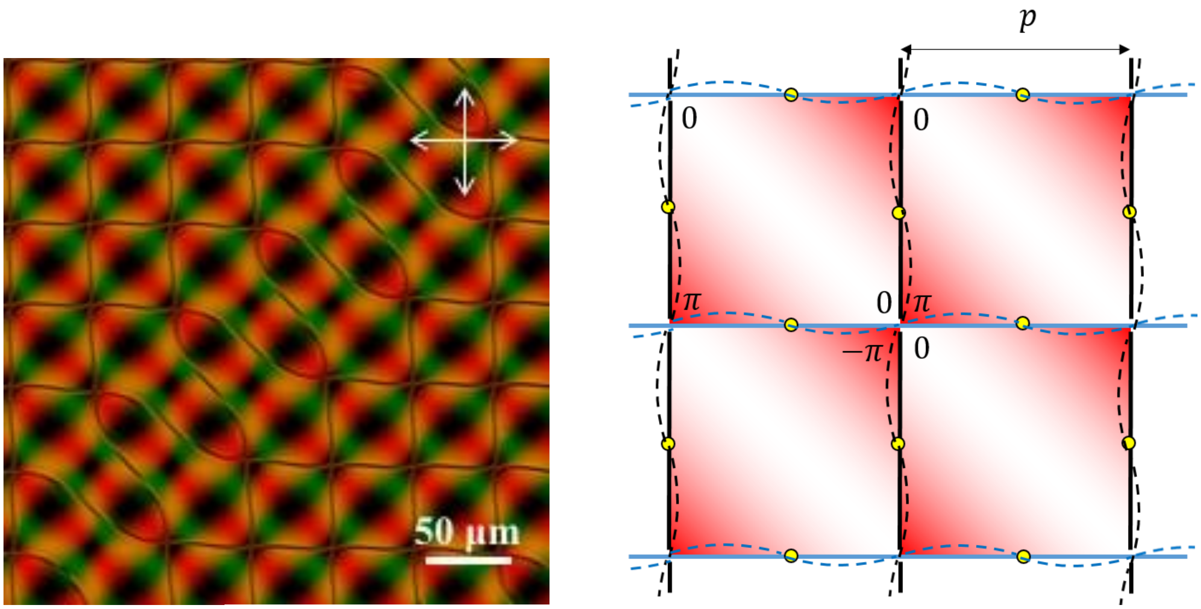

(a) (b)

Supplementary Figure 15: Observation of coexisting square web and diagonal disclination lines. (**a**) Polarized micrograph left lower side of the overlapped region. (**b**) Twist energy density map and the curved disclination lines due to the imbalance of twist deformation energy.

This preparation was more aged and the cell thickness got a little thinner than that shown in Fig. 3 as suggested by the deeper coloration. The narrower cell gap increases the twist deformation energy, which resulted in an appreciable periodic deformation of disclination lines in accordance with the Young-Laplace equation, Supplementary Equation 47. The corner area with zero twist angle pressures the disclination lines to bend outward, and the π twist region is pressed inward as illustrated in Supplementary Figure 15b.
